# Implementing the Risk Identification (RI) and Modified Early Obstetric Warning Signs (MEOWS) tool in district hospitals in Rwanda: a cross-sectional study

**DOI:** 10.1186/s12884-020-03187-1

**Published:** 2020-09-29

**Authors:** Eugene Tuyishime, Honorine Ingabire, Jean Paul Mvukiyehe, Marcel Durieux, Theogene Twagirumugabe

**Affiliations:** 1 Department of Anesthesia, Critical Care and Emergency Medicine, College of Medicine and Health Sciences, University of Rwanda, Kigali, Rwanda; 2 University of Virginia, Charlottesville, USA

**Keywords:** Risk identification, Modified early obstetric warning signs, Early warning system, Maternal morbidity, Quality improvement, Rwanda

## Abstract

**Background:**

Despite reaching Millennium Development Goal (MDG) 3, the maternal mortality rate (MMR) is still high in Rwanda. Most deaths occur after transfer of patients with obstetric complications from district hospitals (DHs) to referral hospitals; timely detection and management may improve these outcomes. The RI and MEOWS tool has been designed to predict morbidity and decrease delay of transfer. Our study aimed: 1) to determine if the use of the RI and MEOWS tool is feasible in DHs in Rwanda and 2) to determine the role of the RI and MEOWS tool in predicting morbidity.

**Methods:**

A cross-sectional study enrolled parturient admitted to 4 district hospitals during the study period from April to July 2019. Data was collected on completeness rate (feasibility) to RI and MEOWS tool, and prediction of morbidity (hemorrhage, infection, and pre-eclampsia).

**Results:**

Among 478 RI and MEOWS forms used, 75.9% forms were fully completed suggesting adequate feasibility. In addition, the RI and MEOWS tool showed to predict morbidity with a sensitivity of 28.9%, a specificity of 93.5%, a PPV of 36.1%, a NPV of 91.1%, an accuracy of 86.2%, and a relative risk of 4.1 (95% Confidential Interval (CI), 2.4–7.1). When asked about challenges faced during use of the RI and MEOWS tool, most of the respondents reported that the tool was long, the staff to patient ratio was low, the English language was a barrier, and the printed forms were sometimes unavailable.

**Conclusion:**

The RI and MEOWS tool is a feasible in the DHs of Rwanda. In addition, having moderate or high scores on the RI and MEOWS tool predict morbidity. After consideration of local context, this tool can be considered for scale up to other DHs in Rwanda or other low resources settings.

**Trial registration:**

This is not a clinical trial rather a quality improvement project. It will be registered retrospectively.

## Background

Although Rwanda reached Millennium Development Goal (MDG) 3 (Promote gender equality and empower women), the maternal mortality rate (MMR) in the country is still high. MMR has been reduced from almost 500 per 100,000 live births in 2010 to approximately 200 per 100,000, but this is still far from the 2030 target of 140 per 100,000 [1].

Globally, 75% of maternal deaths are caused by the following 5 complications: hemorrhage, infections, preeclampsia and eclampsia, obstructed labor, and abortions [2]. This is similar to the situation of Rwanda where these 5 common causes of maternal mortality in Rwanda have remained the same for the last decade [1]. In 2015, Post-Partum Hemorrhage (PPH) and sepsis accounted for 46% of maternal deaths in Rwanda; more than 70% of deaths occurred in teaching and district hospitals, and 64% of deaths occurred during the postpartum period [3].

As in many countries, the hospital system in Rwanda includes District Hospitals (DH, about 40) and central Referral Hospitals (RH, 3). Most maternal deaths occur after transfer of patients with obstetric complications from a DH to a RH [3]. This referral system is associated with delays at each level (DH and RH). This suggests that early recognition of patients at high risk of complications might allow earlier transfer before the development of complications and speed up the access to care at higher level by minimizing delays through easy situation awareness, communication, and decision making among teams. For example, studies done in Ireland and Zimbabwe reported an improvement in the time interval between trigger and antibiotic administration, and pre-operative stabilization of women undergoing caesarean section following the implementation of the Early Warning Signs (EWS) tool [4, 5].

Multiple effective tools exist to identify parturient at risk, and in other countries have been shown to improve outcomes [6–12]. However, these tools have never been tested in Rwanda, where patient populations and structure of healthcare delivery are quite different from the context of the tool validations.

We therefore wished to determine the effectiveness of one comprehensive tool developed to fit the context of DHs of Rwanda, the RI and Modified Early Obstetric Warning Signs (MEOWS) tool (See Tables 1 and 2) [6–12]. This tool is based on the risk factors of hemorrhage and preeclampsia used by [6] in California; the risk factors of sepsis used by NICE in 2015, in UK; and regular assessment of 5 physiologic variables: respiratory rate, pulse rate, blood pressure, temperature and mental state [8].

**Table 1:** The Risk identification (RI) and Modified Early Obstetric Warning Score (MEOWS) tool. Risk identification (RI) tool

Criteria	High risk	Moderate risk	Low risk
**Hemorrhage**	**Recognition:** **-On admission:** 1. Placenta previa, low lying placenta 2. Suspected Placenta accreta or percreta 3. Hematocrit < 30, refusal of transfusion, AND other risk factors: 4. Platelets < 100,000 5. Active bleeding (greater than show) 6. Known coagulopathy	**Recognition:** **-On admission:** 1. Prior cesarean birth(s) or uterine surgery 2. Multiple gestation 3. > 4 previous vaginal births 4. Chorioamnionitis 5. History of previous PPH 6. Large uterine fibroids	**Recognition:** **-On admission** 1. No previous uterine incision 2. Singleton pregnancy 3. < 4 previous vaginal births 4. No known bleeding disorder
	-Evaluate for development of additional risk factors **in labor and postpartum**: • Prolonged 2nd Stage labor • Prolonged oxytocin use • Active bleeding •Chorioamnionitis • Magnesium sulfate treatment	-Evaluate for development of additional risk factors **in labor and postpartum**: • Prolonged 2nd Stage labor: • Prolonged oxytocin use • Active bleeding • Magnesium sulfate treatment	-Evaluate for development of additional risk factors **in labor and postpartum**: • Prolonged 2nd Stage labor • Prolonged oxytocin use: • Active bleeding •Chorioamnionitis • Magnesium sulfate treatment
	**−1 or more high risk criteria: High risk of hemorrhage**	**−1 or more moderate risk criteria: Moderate risk of hemorrhage**	**No moderate or high risk of hemorrhage: Low risk of hemorrhage**
**Conclusion**	**Response:** **-**Consider referral if not in labor -If in labor close monitoring, type and screen, order 2 units of blood, delivery	**Response:** **-**Consider referral if not in labor (clinical judgment) -If in labor close monitoring, type and screen, book 2 units of blood, delivery	**Response:** **-**Standard of care
**Preeclampsia/Eclampsia**	**Recognition:** **CNS:**	**Recognition:** **CNS:**	**Recognition:** **CNS:**
	**Awareness:** unresponsive	**Awareness:** •Agitated/confused • Drowsy • Difficulty speaking	**Awareness:** Alert/oriented
	**Headache:** Unrelieved headache	**Headache:** • Mild headache • Nausea, vomiting	**Headache:** None
	**Vision:** Temporary blindness	**Vision:** Blurred or impaired	**Vision impairment:** None
	**CVS:** SBP: ≥160 DBP: 50–89 HR: 61–110 Chest pain **RS:** RR: < 10 or > 30 **GIT:** Nausea and vomiting Abdominal pain **Renal: u.o in mls:** ≤30 (in 2 h) **Proteinuria:** Not relevant **Platelet:** < 50 **ASAT/ALAT:** > 70 **Cr:** > 1.2 **MgSO4 toxicity:** Respiration < 12	**CVS:** SBP: 140–159 DBP: 50–89 HR: 111–129 Chest pain **RS:** **RR:** 25–30 **GIT:** Nausea and vomiting Abdominal pain **Renal: u.o:** 30–49 **Proteinuria:** • > + 1, • 300 mg/24 h **Platelet:** 50–100 **ASAT/ALAT:** > 70 **Cr:** 0.9–1.1 **MgSO4 toxicity:** Depression of patellar reflexes	**CVS:** SBP: 100–139 DBP: ≥105 HR: > 130 No chest pain **RS:** **RR:**11–24 **GIT:** None None **Renal: u.o:** ≥50 **Proteinuria:** Trace **Platelet:** > 100 **ASAT/ALAT: <** 70 **Cr:** < 0.8 **MgSO4 toxicity:** • DTR + 1 • Respiration 16–20
	**1 or more high risk criteria: High risk** **of preelampsia/eclampsia**	**1 or more moderate risk criteria: Moderate risk of preeclampsia/eclampsia**	**No moderate or high risk criteria: No risk** **of preeclampsia /eclampsia**
**Conclusion**	**Response:** Immediate evaluation (ABCDE approach) • Transfer to higher acuity level • 1:1 staff ratio • Labetalol/hydralazine in 30 min • In-person evaluation • Magnesium sulfate loading or maintenance infusion O2 at 10 L per rebreather mask • R/O pulmonary edema • Chest x-ray •Safe referral to tertiary center	**Response:** •Notify In charge RN or Midwife •In-person evaluation •Order labs/tests •Anesthesia consult •Consider magnesium sulfate •Supplemental oxygen •Physician should be made aware of worsening or new-onset proteinuria	**Response:** Proceed with protocol for normal pregnancy
**Sepsis**	**Recognition for every woman (on admission):** Risk factors: 1.gestational diabetes, diabetes or other comorbidities	**Recognition for every woman (on admission):** Risk factors: 1.gestational diabetes, diabetes or other comorbidities	**Recognition for every woman (on admission):** Risk factors: 1.gestational diabetes, diabetes or other comorbidities
	2.needed invasive procedure such as caesarean section, forceps delivery, removal of retained products of conception within 6 weeks	2.needed invasive procedure such as caesarean section, forceps delivery, removal of retained products of conception within 6 weeks	2.needed invasive procedure such as caesarean section, forceps delivery, removal of retained products of conception within 6 weeks
	3.prolonged rupture of membranes	3.prolonged rupture of membranes	3.prolonged rupture of membranes
	4.continued vaginal bleeding or an offensive vaginal discharge	4.continued vaginal bleeding or an offensive vaginal discharge	4.continued vaginal bleeding or an offensive vaginal discharge
	**Diagnosis criteria** 1.**CNS**: new altered mental state on examination	**Diagnosis criteria** **1.CNS**: History of new altered mental state: ----------	**Diagnosis criteria** No high risk or moderate risk criteria met: --------------
	2.**RS:** RR > 25: --------- or need of FiO2 > 40% to keep Sat > 92%: ---------	2.**RS:** RR > 21–24: ----------	
	3. **CVS:** SBP < 90 mmHg: ------ or HR > 130: -----------	3.**CVS:** SBP:91–100 mmHg: -----or HR: 100–130: ---------	
	4.**Renal:** No urine in 18 h: ------- or if foley catheter U.O < 0.5 ml/kg/h: ----------	4.**Renal:** No urine in 12–18 h: ----------- or if foley catheter U.O: 0.5–1 ml/kg/h: --------------	
	5.**Temperature > 39 °C: --------------** 6.**Skin:** Mottled appearance, Cyanosis of skin, lips or tongue, Non-blanching rash of skin: ----------------	5.**Temperature < 36 °C: --------** 6.**Skin:** Signs of potential infection, including redness, swelling or discharge at surgical site or breakdown of wound: --------	
	**−1 or more high risk criteria: High risk of sepsis**	**−1 or more moderate risk criteria: Moderate risk of sepsis**	**-no high or moderate risk criteria: Low risk of sepsis**
**Conclusion**	**Response:** -Immediate review by senior clinical decision maker (ABCDE approach) -Blood test: -Blood gas for glucose and lactate. -Blood culture· -Full blood count· -C-reactive protein· -Urea and electrolytes· -Creatinine· -Clotting screen	**Response:** -Blood test: -Blood gas for glucose and lactate· -Blood culture· -Full blood count· -C-reactive protein· -Urea and electrolytes· -Creatinine· -Clotting screen -Review by senior clinical decision maker within 1 h -IV antibiotics within 1 h − 500 ml bolus every 15 min, repeat up to 3 times -If no definitive condition identified, repeat structured assessment at least **hourly**	**Response:** -Clinical assessment and manage according to clinical judgement
	- **MEOWS** -IV antibiotics within 1 h − 500 ml bolus every 15 min, repeat up to 3 times, if SBP < 90 mmHg give adrenaline 1 mg/500 ml NS to keep MAP> 65 or SBP > 90 -Refer to a tertiary hospital	**- MEOWS** -Source control within 6 h, if deep infection refer to a tertiary hospital

**Table 2:** The Risk identification (RI) and Modified Early Obstetric Warning Score (MEOWS) tool. Modified Early Obstetric Warning Score (MEOWS) tool

Score	3	2	1	0	1	2	3
Temperature		< 35°. C		35–37.4°. C		37.5–39°. C	> 39°. C
Systolic * BP	≤70	71–79	81–89	90–139	140–149	150–159	≥160
Diastolic * BP			≤45	46–89	90–99	100–109	≥110
Pulse		≤ 40	40–50	51–100	101–110	111–129	≥ 130
Respiratory Rate		≤ 8		9–14	15–20	21–29	≥30
AVPU				Alert	Responds to Voice	Responds to Pain	Unconscious
Urine output mLs/hr	< 10	< 30		Not Measured			

If the pulse rate is higher than the systolic blood pressure then score 2 for ‘Pulse’

MEOWS less or equal to 2: Current plan

MEOWS =3–5: Repeat observations, Senior midwife to review, Medical review

MEOWS high or equal to 6: Inform Coordinator or Senior Midwife, Medical review, Anesthesia review, Referral

Our study had as primary objective to determine if the use of the RI and the MEOWS tool is a feasible intervention in the setting of DH in Rwanda.

Our secondary goals were to test for association between abnormal RI and MEOWS score and presence of morbidity, and to evaluate the participants’ experience during the use of the RI and MEOWS tool.

## Methods

### Aim

This study aimed to evaluate the feasibility of implementing the risk factors identification and MEOWS tool in the setting of DH in Rwanda.

### Setting

This study was conducted in 4 DH referring to the 2 main RH in Rwanda: the Centre Hospitalier Universitaire de Kigali (CHUK) and the Centre Hospitalier Universitaire de Butare (CHUB). The DH in the study were at Nyanza, Kabutare, Muhima, and Kibagabaga. They are located within 1 h drive to the Referral hospitals and have a large number of deliveries (Table 3). They were selected to provide representative examples of typical DHs in various parts of the country.

**Table 3:** Characteristics of the 4 district hospitals involved in the implementation of the RI and MEOWS study

Criteria	Nyanza	Kabutare	Muhima	Kibagabaga
Number of maternity staff				
Midwifes	13	15	48	46
General practitioners	9	3	17	19
Non physician anaesthetists	4	5	8	9
Obstetricians	1	0	2	2
Paediatricians	2	1	4	2
Average number of deliveries per month				
Vaginal deliveries	152	163	505	500
Caesarean sections	133	105	178	200
Total	285	268	683	700

### Study design

This was a cross-sectional study conducted in 4 district hospitals (Table 3) using survey methodology.

To assess our primary objective, we collected data on feasibility (completeness rate) during the use of the RI and MEOWS tool (how often and how completely the tool was actually used). In addition, staff were interviewed about their experience while using the RI and MEOWS tool and ability to incorporate it into their workflow.

To assess our secondary objective, we collected clinical data during the implementation period to test for association between abnormal RI and MEOWS score and presence of morbidity as measured a composite outcome of infection, hemorrhage and pre-eclampsia by calculating the relative risk. Also, in order to evaluate the usefulness of the RI and MEOWS tool, we calculated its sensitivity, specificity, accuracy, positive predictive values, and negative predictive values.

Our patient sample size included all parturient presenting at the hospitals between January 1, 2019 and June 30, 2019.

### Intervention

From January to March 2019, the RI and MEOWS tool was adapted to Rwanda context using a modified Delphi method, where a team of 2 anesthesiologists and 2 senior anesthesia residents developed suggested changes to fit the context of DHs in Rwanda.

The main changes were related to the availability of laboratory tests, the different healthcare providers, and the structure of the Rwandan referral system (Tables 1 and 2).

From March to June 2019, the research team implemented the RI and MEOWS tool (Tables 1 and 2). For each hospital, the research team conducted a 20 min teaching session explaining use of the RI and MEOWS tool to all maternity staff during the regular morning meeting. In addition, a co-investigator (HI) selected one coach per hospital to ensure the availability of printed forms in each patient’s file and to provide mentorship to all maternity staff as needed. The coach was also available to support both the staff during the use of RI and MEOWS tool and the data collection team. Furthermore, the research team provided needed remote mentorship to each coach through regular phone calls and WhatsApp messages.

### Statistical analysis and sample size calculation

Our primary endpoint was the fraction of parturient for which the RI and MEOWS tool was fully completed and number of staff that felt it was acceptable as a tool to include in their workflow. Descriptive statistics were used, we reported frequencies and percentages for categorical data, and mean and standard deviation ranges continuous data.

For the secondary outcomes, we tested for association between abnormal RI and MEOWS score at admission and presence of morbidity at discharge by calculating relative risk for a composite outcome of infection, hemorrhage and pre-eclampsia. All statistical tests, we regarded a value of *p* <  0.05 as statistically significant.

Sensitivity, specificity, positive predictive values, and negative predictive values were calculated for the sample. SPSS version 2013 was used for analysis.

As a similar study done in UK had a sample size of 676 [13]. In order to have an adequate sample we recruited patients from 4 district hospitals conducting at least 250 deliveries each month.

## Results

Table 3 describes the capacity (number of staff and deliveries) of the 4 district hospitals selected to be included into our study.

Table 4 describes the completeness of the RI and MEOWS tool. Among 478 forms used, 363 (75.9%) forms were fully completed, 79 (16.5%) partially completed, and 36 (7.5%) were not completed at all.

**Table 4:** Patients’ demographics, completeness of the use of the RI and MEOWS tool, and outcome, N: 478

Variable	Number (%)
Age (Mean, SD)	28.30, 6.38
Gravida (Mean, SD)	2.58, 1.91
Parity (Mean, SD)	1.43, 1.67
ANC (Mean, SD)	2.83, 1.15
Married	
Yes	420 (89.0)
No	52 (11.0)
Insurance	
Yes	450 (95.1)
No	23 (4.9)
Social category	
1	37 (15.7)
2	82 (34.9)
3	115 (48.9)
4	1 (0.4)
District hospital	
Kibagabaga	135 (28.2)
Muhima	136 (28.5)
Kabutare	139 (29.1)
Nyanza	65 (13.6)
Tool use	
Completed	**363 (75.9)**
Partially completed	79 (16.5)
Not completed	36 (7.5)
Morbidity	
Yes	49 (10.3)
No	429 (89.7)
Length of stay (Mean, SD)	3.05 (2.08)
Outcome	
Referral	11 (2.3)
ICU	7 (1.5)
Reoperation	2 (0.4)
Care at DH	458 (95.8)

Tables 5 and 6 describe the experience of staff during the use of the RI and MEOWS tool. When asked about their experience during use of the RI and MEOWS tool, most of the respondents reported that the tool was easy or very easy to use (92%), they were willing to use the tool regularly (90.9%), the tool had improved awareness of patient safety (91.3%), and the tool decreased delay in recognition and management of critically ill obstetric patients (86.4%).

**Table 5:** Respondents’ demographics and experience during use of the RI and MEOWS tool. Respondents’ demographics

Demographics	Number (%)
Hospital name	
Kibagabaga	14 (56)
Kabutare	11 (44)
Profession	
Midwife	23 (92)
Nurse	2 (8)
Experience	
< 1	8 (32)
2–4	9 (36)
5–7	6 (24)
8–10	1 (4)
> 10	1 (4)

**Table 6:** Respondents’ demographics and experience during use of the RI and MEOWS tool. Respondents’ experience during use of the RI and MEOWS tool

Questions	Responses			
How do you think using the risk factors identification and MEOWS tool within the existing patient file was?	Very difficult 0 (0%)	Difficult 2 (8%)	Easy 16 (64%)	Very easy 7 (28%)
To what extent are you willing to use regularly the Risk identification and MEOWS tool to your facility?	Very resistant 0 (0%)	Resistant 2 (9.1)	Willing 9 (40.9)	Very willing 11 (50%)
To what extent do you believe use the risk identification and MEOWS tool has improved awareness of patient safety at your health care facility?	Not at all 0 (0%)	Somewhat significant 2 (8.7%)	Significant 9 (39.1%)	Very significant 12 (52.2%)
To what extent do you believe use of the Risk identification and MEOWS tool has decreased delay in recognition and management of critically ill obstetric patients to your facility?	Not at all 0 (0%)	Somewhat significant 3 (13.6%)	Significant 4 (18.2%)	Very significant 15 (68.2%)

When asked about challenges faced during use of the RI and MEOWS tool, common responses included that the tool was long, it was difficult to use with a low staff to patient ratio, English language was a barrier, and there was unavailability of printed forms.

Tables 7 and 8 describe the capacity of the RI and MEOWS tool to predict morbidity. Among 478 forms within patients’ charts, only 399 had complete data on outcomes of interest (RI and MEOWS tool scores and morbidity) and were considered for analysis. The results showed that the RI and MEOWS tool predicts morbidity with a sensitivity of 28.9%, a specificity of 93.5%, a PPV of 36.1%, a NPV of 91.1%, an accuracy of 86.2%, and a relative risk of 4.1 (95% Confidential Interval (CI), 2.4–7.1).

**Table 7:** Comparison of RI and MEOWS tool scores (Moderate/High versus Low) and Morbidity (Yes versus No), N: 399. Cross tabulation of RI and MEOWS tool scores and Morbidity

	Morbidity: Yes	Morbidity: No
**RI & MEOWS level: Moderate or High**	13	23
**RI & MEOWS level: Low**	32	331

**Table 8:** Comparison of RI and MEOWS tool scores (Moderate/High versus Low) and Morbidity (Yes versus No), N: 399. The characteristics of the RI and MEOWS tool

RI & MEOWS level	Chi-Square (*P* value)	RR (95% CI)	Sensitivity	Specificity	Accuracy	PPV	NPV
Moderate or High Low	< 0.0001	**4.1 (2.4–7.1)**	**28.9%**	**93. 5%**	**86.2%**	**36.1%**	**91.1%**

Morbidity: defined as a composite outcome of PPH, Preeclampsia or Infections**,**

*PPV* Positive predictive value, *NPV* Negative Predictive Value

## Discussion

The completion of the RI and MEOWS tool by 75.9% of participants suggests an adequate feasibility. Our result was consistent with other previous studies although the level of completeness of our study was not as substantial as in other studies like the study done in UK, Ireland, and Zimbabwe that reported an improvement in the frequency of documentation of vital signs, the time interval between trigger and antibiotic administration, and pre-operative stabilization of women undergoing caesarean section following the implementation of the Early Warning Signs (EWS) tool [4, 5, 11].

In addition, our study found that the abnormal RI and MEOWS tool predicted morbidity (*P* <  0,0001) with a low sensitivity of 28.9%, a high specificity of 93.5%, a high accuracy of 86.2%, a low positive predictive value of 36.1%, and a high negative predictive value of 91.1%.

These findings are similar to most results from other multiple studies conducted in different settings. For example, Singh S et al., [13, 14], did 2 studies implementing the MEOWS with more than 1600 patients in total; the results showed a high sensitivity (89%) and (86.4%), high specificity (79%) and (85.2%), an acceptable PPV (39%) and (53.9%), and a high NPV (98%) and (96.9%) for both studies respectively [13, 14]. The significant difference between our study and the studies done by Singh et al. is a low sensitivity. This can be explained by the fact that, in our context, some patients may develop direct complications of pregnancy like PPH without other risk factors especially when procedures are performed by non-specialists.

When asked about challenges faced during use of the RI and MEOWS tool, most of the respondents reported that the tool was long, the staff to patient ratio was low, the English language was a barrier, and the printed forms were sometimes unavailable. Despite facing these challenges, two essential actions led to a successful implementation of the RI and MEOWS. Those actions include adding the RI and MEOWS tools within patients’ charts and nominating one Coach per site to provide regular support to local staff.

There are other challenges to be considered for the successful implementation of the MEOWS tool which have been reported in the literature. These include the lack of multidisciplinary coordination and buy-in, inadequate education about the tool, suboptimal integration within the hospital culture, lack of leadership support, and suboptimal alignment with other quality improvement projects [15–20].

Furthermore, our study found a relative risk of 4.1 (95% CI, 2.4–7.1) suggesting that having moderate or high scores on the RI and MEOWS increases risk of morbidity by 4 fold. This can help timely triaging of high-risk patients with potential to improve outcomes.

Similarly, the implementation of the Obstetric EWS has been found to be effective in predicting severe morbidity, to contribute to improved quality of care, to prevent progressive obstetric morbidity and to improve health outcomes [21]. However, there is limited evidence of the effectiveness of the Obstetric EWS in reducing maternal death across all settings [21].

There are several limitations to consider while interpreting the results of this study. Firstly, our study was conducted in only 4 district hospitals and the results and conclusions may not be applicable to other hospital settings. These hospitals, however, are representative of the country of Rwanda, and the results of this study could be applied to the remaining hospital systems within this country and similar other countries. Secondly, the sample size was small; the study was not powered to determine a difference in mortality.

## Conclusion

The RI and MEOWS tool is a feasible and acceptable in the DHs of Rwanda. In addition, having moderate or high scores on the RI and MEOWS tool predict morbidity. After consideration of local context, this tool can be considered for scale up to the rest of district hospitals of Rwanda or other low resources settings. Further studies are needed to evaluate the impact of the RI and MEOWS tool on maternal mortality in low resources settings.

## Data Availability

The datasets used and/or analyzed during the current study are available from the corresponding author on reasonable request.

## Abbreviations

RI and MEOWS: Risk Identification and Modified Early Obstetric Warning Signs
MMR: Maternal Mortality Rate
MDG: Millennium Development Goal
UK: United Kingdom
MOH: Ministry of health
DH: District Hospital
RH: Referral Hospital
CEMACH: Confidential Enquiry into Maternal and Child Health
CMQCC: California Maternal Quality Care Collaborative
NICE: National Institute for Health and Care Excellence
CHUK: University Teaching Hospital of Kigali
CHUB: University Teaching Hospital of Butare
SPSS: Statistical Package for the Social Sciences
WFSA: World Federation Society of Anesthesiologists
